# Mechanical characterization of the P56 mouse brain under large-deformation dynamic indentation

**DOI:** 10.1038/srep21569

**Published:** 2016-02-22

**Authors:** David B. MacManus, Baptiste Pierrat, Jeremiah G. Murphy, Michael D. Gilchrist

**Affiliations:** 1 School of Mechanical & Materials Engineering, University College Dublin, Dublin, Ireland; 2 Department of Mechanical & Manufacturing Engineering, Dublin City University, Dublin, Ireland

## Abstract

The brain is a complex organ made up of many different functional and structural regions consisting of different types of cells such as neurons and glia, as well as complex anatomical geometries. It is hypothesized that the different regions of the brain exhibit significantly different mechanical properties, which may be attributed to the diversity of cells and anisotropy of neuronal fibers within individual brain regions. The regional dynamic mechanical properties of P56 mouse brain tissue *in vitro* and *in situ* at velocities of 0.71–4.28 mm/s, up to a deformation of 70 μm are presented and discussed in the context of traumatic brain injury. The experimental data obtained from micro-indentation measurements were fit to three hyperelastic material models using the inverse Finite Element method. The cerebral cortex elicited a stiffer response than the cerebellum, thalamus, and medulla oblongata regions for all velocities. The thalamus was found to be the least sensitive to changes in velocity, and the medulla oblongata was most compliant. The results show that different regions of the mouse brain possess significantly different mechanical properties, and a significant difference also exists between the *in vitro* and *in situ* brain.

Traumatic brain injury (TBI) is a major cause of death and disability worldwide, accounting for 1.7 million injuries, 275,000 hospitalizations, and 50,000 deaths in the United States each year^1^. In Europe, injuries are the leading cause of death between the ages of 15 and 44, with road accidents in Southern Europe and falls related to alcohol consumption in Northern Europe constituting the majority of these cases, and with head trauma accounting for the majority of trauma related deaths^2^. The high occurrence, physical and socioeconomic impact of TBIs^3^ has meant that it is an important focus area for research with many laboratories creating detailed 3D Finite Element models of the head and brain^4^,^5^,^6^,^7^. These models are used to investigate the effects that stresses and strains have on the brain and to identify which regions of the brain are subjected to maximum values of stress and strain. However, the brain is a complex organ made up of many different functional and structural regions consisting of different types of cells such as neurons and glia, as well as complex anatomical geometries^8^. It is hypothesized that the different regions of the brain exhibit significantly different mechanical properties, which may be attributed to the diversity of cells and anisotropy of neuronal fibers within individual brain regions. Current computer simulation models lack accurate constitutive data of the brain’s individual regions at dynamic strain rates that are comparable to those experienced during traumatic events. This data is required to correctly predict the stresses and strains produced in the brain during these scenarios, and to anticipate which regions are most susceptible to mechanical damage.

The current research presents tissue-level hyperelastic constitutive data for the mouse cortex, cerebellum, thalamus, and medulla, under large deformation dynamic indentation using a custom-built micro-indentation apparatus (Fig. 1). Indentation sites are highlighted in Fig. 2. Hyperelastic models are used to determine the nonlinear force-displacement response of mouse brain tissue at different velocities, using an inverse Finite Element (FE) approach (Fig. 3), hyperelastic data reported here can be used with existing viscoelastic data to model mouse brain tissue at these deformation rates. Indentation presents as an ideal loading modality for studying brain trauma. The stress fields which arise in the tissue due to these indentations are a combination of compressive, tensile and shearing forces, and are similar to those which occur during head impacts^9^,^10^,^11^,^12^,^13^ and to the forces applied to the brain by surgical equipment. However, there has been little published work on the micro-scale properties of brain tissue using the micro-indentation method to characterize the local hyperelastic response of different brain regions at dynamic rates.

Here, the first mechanical characterization study of the dynamic, hyperelastic response for different regions of the P56 mouse brain, under large-deformation indentation, *in vitro* and *in situ*, is presented. Indentations are performed at velocities ranging from 0.71–4.28 mm/s, comparable to those in both surgical and traumatic brain injury cases. The force-displacement data recorded from experiments is fit to three hyperelastic material models (Neo-Hookean, Mooney-Rivlin, and Ogden) using the inverse Finite Element method. These constitutive models were chosen based on their widespread use in the biomechanics community^10^,^11^,^14^,^15^,^16^,^17^,^18^, and ease of implementation in Finite Element programs. The models use a strain energy density function to relate stress and strain. The Neo-Hookean model uses the material parameter C_10_, where *μ* = 2C_10_, and the Mooney- Rivlin model uses parameters C_10_ and C_01_, where *μ* = 2(C_10_ + C_01_), and *μ* is the shear modulus. The shear modulus is used to interpret the rigidity of a specimen and can be used in material models to describe the deformation process of a material under applied forces. For an in-depth discussion of elasticity and hyperelastic models applied to soft tissue mechanics, readers are referred elsewhere^10^,^11^,^14^,^19^,^20^. The material parameters reported here, together with previously reported viscoelastic data^10^, can be implemented into computer models of the mouse head and brain to provide an approximation of the effect that trauma has on the individual regions of the organ.

## Results

### Force-displacement behavior of mouse brain tissue

A total of 180 mice were used in this study; ten mice were used for each velocity and region. Indentations were carried out at velocities of 0.71, 2.14, and 4.28 mm/s on the cerebral cortex, cerebellum, thalamus, and medulla oblongata of P56 mixed sex mice. These locations were chosen due to their vital physiological and psychological significance for quality of life, and to account for regional differences in brain tissues mechanical properties. Independent indentations were also performed on the cortex and cerebellum *in situ* to investigate if differences between *in vitro* and *in situ* tissue exist. Data from force-displacement curves were analysed up to a 70 μm displacement for each experiment (Fig. 4) to investigate the nonlinear loading response of brain tissue. The results show an increase in reaction force of the tissue with increasing velocity which highlights the rate dependent properties of the tissue. Each region demonstrates its own unique rate effect response, with the medulla being most sensitive to changes in velocity and the thalamus being least sensitive. The medulla was indented perpendicular to the fiber direction and was found to be the most compliant of the brain regions measured. The *in situ* cortex was less sensitive to changes in velocity and produced larger reaction forces at each velocity when compared with the *in vitro* cortex. Conversely, the *in situ* cerebellum exhibits a stronger rate effect compared with the *in vitro* cerebellum; however, the reaction forces are comparable at each velocity. It is evident from the force-displacement curves that each of the regions investigated have unique responses to the mechanical forces, and also to the rate of these applied forces, suggesting that some regions of the brain may be more susceptible to trauma than others.

### Mechanical characterization of mouse tissue, *in vitro*

The force-displacement data from experiment were fit to three hyperelastic material models, Neo-Hookean, Mooney-Rivlin, Ogden, for each region and each velocity using the inverse Finite Element method. The tissue’s shear modulus for all regions increased with an increasing velocity, demonstrating a rate dependence property of the tissue. The cerebral cortex has shear moduli values of 2.22, 5.26, and 6.72 kPa at 0.71, 2.14, and 4.28 mm/s (Fig. 5a) respectively, corresponding to an overall increase of 67% between the lowest and highest velocities. The cerebellum, thalamus, and medulla experience total increases of 56%, 11%, and 71% in apparent shear moduli, respectively. Table 1 presents the material parameters for the three hyperelastic material models to which the data was fit; Neo-Hookean, Mooney-Rivlin, and Ogden. The data in Table 1 is *in vitro* data unless otherwise stated. Each of the models achieved an excellent fit with the data, R^2^ > 0.9 for all models. However, the Ogden model achieved a marginally better fit than the others. This is due to the nonlinear parameter, α, which accounts for the nonlinearity of the force-displacement curves. Most regions fit with the Ogden model achieved an R^2^ value of 0.999. The model with the lowest R^2^ value (0.976) was for the 4.28 mm/s cortex data when fit to the Mooney-Rivlin model. ANOVA with a post hoc test was carried out to compare the shear moduli values of each region to determine whether a statistically significant difference exists between the brain regions. A significant difference was found between the regions investigated and is reported in Fig. 5b. *In vitro* regions which were not significantly different comprised of the cerebellum and thalamus at all velocities, the medulla and thalamus at 2.14 and 4.28 mm/s, and the cerebellum and medulla at 4.28 mm/s. All other regions were significantly different for all velocities, *in vitro*, satisfying the hypothesis that brain regions possess their own unique mechanical properties.

### Mechanical characterization of mouse tissue, *in situ*

The *in situ* force-displacement curves were fit to the same hyperelastic material models as the *in vitro* data. However, only the cortex and cerebellum regions could be investigated using this methodology. There is an apparent difference between the mechanical response of *in situ* and *in vitro* brain tissue, which is most notable and has previously been reported for the cortex^21^,^22^,^23^, but to the best of the authors knowledge there has been no reported data on *in situ* cerebellum tissue.

The cortex tissue experiences an increase in apparent shear modulus of 51%, 20%, and 10% between *in vitro* and *in situ* environments at 0.71, 2.14, and 4.28 mm/s, respectively. Conversely, the cerebellum initially exhibits a 70% increase in shear modulus between the *in vitro* and *in situ* cases for 0.71 mm/s. However, a reduction of 8% and 5% of the apparent shear moduli is experienced by the cerebellum at 2.14 and 4.28 mm/s, respectively.

Table 1 contains the mechanical properties of the *in situ* cortex and cerebellum for the Neo-Hookean, Mooney-Rivlin, and Ogden hyperelastic material models. Overall, each model achieved an excellent fit with the experimental data; the lowest R^2^ value was for the cerebellum with a value of 0.990 for the 4.28 mm/s Neo-Hookean data. The differences in apparent shear moduli for given velocities is presented graphically in Fig. 5a. Figure 5b presents the statistically significant differences between the regions investigated. For the *in situ* data, only the 0.71 mm/s *in situ* cortex was significantly different from the *in vitro* cortex data. The *in situ* cortex was significantly different from the *in situ* cerebellum and all other *in vitro* regions for each velocity. The *in situ* cerebellum was also significantly different compared with the *in vitro* cerebellum data for 0.71 mm/s. It is evident from these graphs (Fig. 5a,b) that the different regions of the brain possess significantly different mechanical properties.

## Discussion

The current study presents the local mechanical properties of *in vitro* and *in situ* P56 mouse brain tissue at dynamic rates that have been hypothesized to cause injury, i.e. >10/s strain-rate and >10% strain^10^. Micro-indentation experiments were carried out on the cortex, cerebellum, thalamus, and medulla oblongata (Fig. 2). Velocities in the range 0.71–4.28 mm/s were applied locally to these regions to examine the regional differences in mechanical properties to the application of dynamic loading. The resulting shear moduli of the tissue increases with increasing velocity as shown in Fig. 5a which is consistent with previous studies^10^,^11^,^14^. For the *in vitro* study, it was found that each region had a unique response to the applied mechanical load with the cerebral cortex being the stiffest brain region investigated with values consistent of those reported previously for *in vivo* mouse cortex^24^. The thalamus was more compliant than the cortex and cerebellum but was stiffer than the medulla, which was the most compliant of the regions investigated, which is in agreement with previous results reported on the rat and porcine brains^25^,^26^. However, the cerebellum was found to be relatively stiffer in the mouse model presented here, compared to the porcine and rat models^25^,^26^, and less stiff relative to the cortex which is consistent with the viscoelastic properties of human cerebellum and cortex data reported previously^27^. The relative regional stiffness of the mouse brain is comparable to those of the rat brain reported by Finan *et al.*^28^, with the cortex being one of the stiffest regions, the thalamus was found to be less stiff than the cortex but stiffer than the medulla (brainstem), consistent with the results reported here. However, in the rat model, the cerebellum is reported as the most compliant region, whereas for the mouse model it is the medulla that was found to be the softest region. Also, previous studies have shown the thalamus to elicit a stiffer response compared with the cortex in the porcine and human brain^29^,^30^ which is inconsistent with mouse and rat data. The medulla was also found to be the most sensitive to changes in velocity with an overall increase in shear modulus of 71%, whereas the thalamus was found to be the least sensitive to changes in velocity with an overall increase in shear modulus of 11% between 0.71 and 4.28 mm/s. This might be a feature of slicing the brain tissue which is required to access this region, whereas all other regions were indented on their exterior surfaces. However, this currently suggests that the thalamus is weakly viscoelastic and should be investigated further in the context of its mechano-pathophysiology. The thalamus’ low rate-effect is especially significant in the context of repetitive concussion, as discussed by Smith *et al.*^31^. The data reported here, presented in Table 1, supports the hypothesis that different regions in the brain possess significantly different mechanical properties (Fig. 5) and elicit their own unique responses to the application of mechanical forces (Fig. 4).

The *in situ* cortex tissue (4.58 ± 0.28, 6.60 ± 0.17, 7.52 ± 0.16 kPa) was found to be stiffer than the *in vitro* tissue (2.22 ± 0.12, 5.26 ± 0.32, 6.78 ± 1.28 kPa) for all velocities, which agrees with Prevost *et al.*^21^, and Gefen *et al.*^22^. It is hypothesized that the higher values of cortex shear moduli *in situ* are caused by residual stresses in this region of the brain^32^ which are released upon the removal of the boundary condition applied by the skull. Conversely, for the cerebellum there was an insignificant difference between the *in vitro* and *in situ* cases suggesting that this region of the mouse brain has less residual strain. The mean moduli values of the *in situ* cerebellum (1.90 ± 0.58, 2.16 ± 0.74, 2.26 ± 1.90 kPa) were found to be less than those of the *in vitro* cerebellum (1.34 ± 0.22, 2.36 ± 0.72, 2.38 ± 0.34 kPa) for 2.14 and 4.28 mm/s, which is inconsistent with previous reports in the literature for cortex tissue^21^,^22^. However, these differences between *in vitro* and *in situ* cerebellum were found to be significant at the low velocity (0.71 mm/s) only (Fig. 5b).

The shear moduli and rate-effect results reported here could offer insight into how traumatic brain injury affects certain regions more than others in a mouse model of TBI such as those described previously^33^. It is evident from the data provided in Table 1 and Fig. 5 that the regions of the mouse brain exhibit regionally unique mechanical properties. A possible explanation for the differences in mechanical properties of brain tissue is the underlying cellular structure of these regions. Azevedo *et al.* have shown that the human cortex contains approximately 3.76 times more non-neuronal cells than neuronal cells, whereas the cerebellum contains 4.3 times more neuronal cells to non-neuronal cells, and the rest of the brain (RoB) contains 11.35 times more non-neuronal cells to neuronal cells^34^. Differences in the mechanical properties of these cells^35^,^36^ and how they interact with each other may offer a mechanical explanation for the regionally varying material properties of brain tissue. Further analysis of the distribution and density of cell types within specific brain regions is required for a more complete understanding of this as there is limited data on the number of cells and cell type in each region, such as neurons, astrocytes, GFAP-microglia, and oligodendrocytes.

While this research provides an advancement in the understanding of how different regions of the mouse brain respond to mechanical trauma, there are limitations to this work which may account for some of the discrepancies with others reported in the literature such as the use of a square cross-sectional indenter to indent the samples instead of a spherical or cylindrical punch. An axisymmetric probe would allow the use of an axisymmetric FE model which would decrease the computation time taken to perform the inverse FE procedure. Fresh human tissue is the ideal sample for determining the material properties of brain tissue under traumatic loading. However, the difficulties involved with obtaining fresh human tissue, due to increased post-mortem time, diseased, and elderly tissue samples, the obtained human tissue is usually not in an ideal state to quantify the true mechanical properties. The experimental temperature, 22 °C, used here may have an impact on the reported mechanical properties^37^, an experimental temperature of 37 °C may be more suitable but would need to be correlated with *in vivo* results to ensure that post-mortem effects don’t significantly alter the mechanical properties of *in vitro* tissue, even those measured at 37 °C. It has recently been suggested that the Neo-Hookean, and Mooney-Rivlin model employed here may not be adequate to accurately model brain tissue^38^ and that 6 or 8 term Ogden models may be more suitable for multi-modal deformation (compression-tension). However, the data presented here is uni-modal indentation, and the R^2^ values in Table 1 suggests that the current models used here are adequate to describe the behavior of mouse brain tissue under indentation up to 70 μm, and velocities between 0.71–4.28 mm/s, with overall R^2^ values of 0.996, 0.996, 0.998, for the Neo-Hookean, Mooney-Rivlin, and Ogden models, respectively.

The current research has, for the first time, characterized the dynamic mechanical properties of P56 mouse cortex, cerebellum, thalamus, and medulla, under large deformation. The mechanical properties of *in situ* cortex, and for the first time *in situ* cerebellum, have also been reported, and statistically significant differences between mouse brain regions have been established. We have used the inverse Finite Element method to fit the primary experimental data to three hyperelastic models, the Neo-Hookean, Mooney-Rivlin, and Ogden model, which have been shown to be capable of accurately describing the uni-modal deformation of brain tissue under large deformation indentation (mean R^2^ for all data = 0.997). The mechanical properties provided for these regions can be implemented immediately in current and next generation Finite Element models of the mouse brain with existing viscoelastic data of brain tissue^10^,^11^,^14^ to provide a qualitative approximation of how brain tissue may respond in traumatic impact injury events and neurosurgical scenarios. In order to fully describe the response of brain tissue in traumatic events both the loading and relaxation curves are required. The data presented here provides an in depth analysis of dynamic loading response of different regions of the mouse brain under large-deformation indentation. Future work will focus on coupling the hyperelastic loading and viscoelastic relaxation behavior of brain tissue to determine the time-dependent properties of the individual regions, and if a significant difference exists between them as has been shown here for the hyperelastic material parameters.

## Methods

### Experimental setup

**Figure 1:**
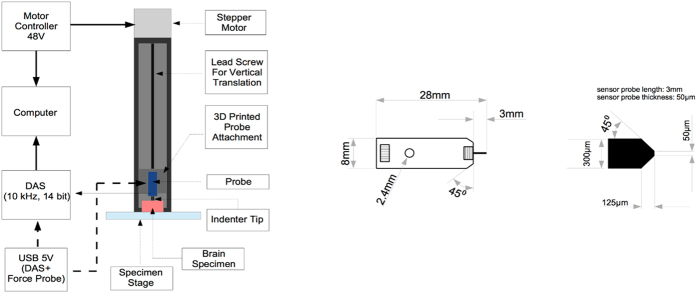
(**a**) Schematic Diagram of Test Apparatus, (**b**) FemtoTools FTS-10000 Microforce Sensing Probe, detailed in MacManus (2015)^39^.

**Figure 2:**
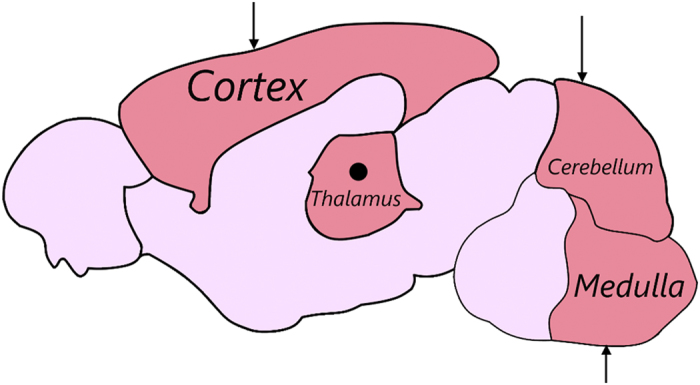
Sagittal section showing regions and Indentation Sites of the Mouse Brain. Indentations were performed to a depth of 70 μm at 0.71, 2.14, and 4.28 mm/s, on the surfaces of the cerebral cortex, cerebellum, and medulla oblongata. The mouse brain was then sectioned through the midline fissure to expose the thalamus tissue for indentation. The arrows indicate the direction that the indentation was performed for the cortex, cerebellum, and medulla oblongata. The thalamus was indented in the sagittal plane.

**Figure 4:**
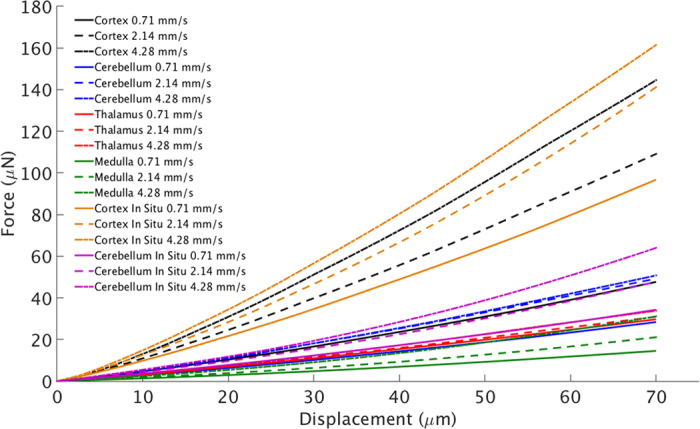
Force-displacement curves of the *in vitro* cortex, cerebellum, thalamus, and medulla, at 0.71, 2.14, and 4.28 mm/s. Also shown are the individual force-displacement curves for the *in situ* cortex and cerebellum for the same velocities. The different responses of the individual regions to mechanical loads are evident from this graph. What is most notable is the significantly larger forces recorded from the *in vitro* and *in situ* cerebral cortex at higher velocities compared with the other regions investigated.

**Figure 5:**
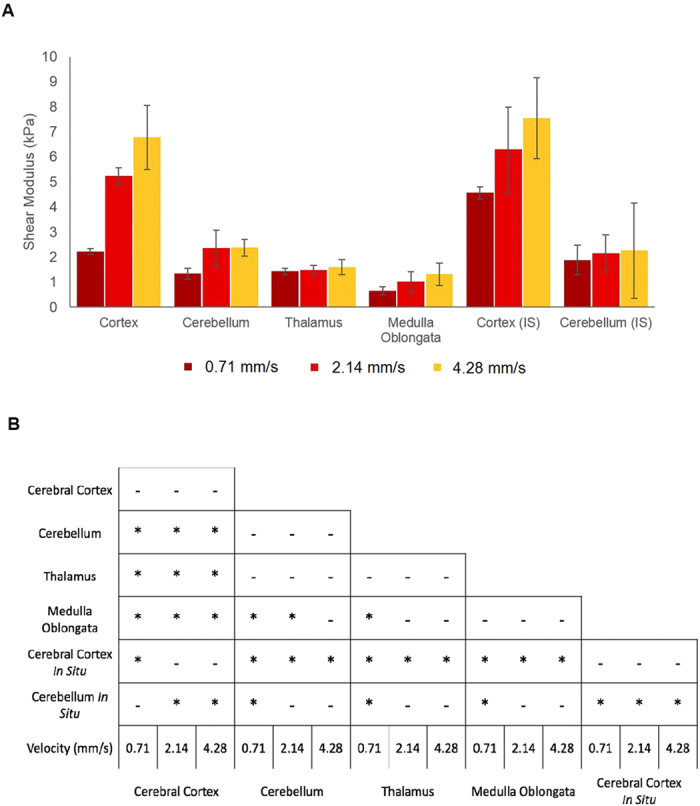
(**A**) Comparison of shear modulus for thalamus, medulla, and *in vitro* and *in situ* (IS) cortex and cerebellum. Each regions unique material properties are clearly presented here. Also evident is the difference of mechanical properties between the *in vitro* and *in situ* tissues under dynamic-rates. (**B**) *represents a statistically significant result from ANOVA post-hoc tests, - represents an insignificant difference. Each region is compared with the other regions for corresponding velocities. Significant differences between velocities in the same region are not presented.

A custom-built micro-indentation device was developed to investigate the local mechanical properties of brain tissue under dynamic loading conditions, (Fig. 1a)^39^. The device consists of three major components, the 3D-printed force-sensing probe stage, the linear stage (ZABER-LSQ), which moves the force-sensing probe stage in the vertical direction at speeds reaching 1 m/s, and a FemtoTools STS-10000 force sensing probe which is used to measure the reaction forces of the tissue with a 10 kHz sampling rate (Fig. 1b). The probe tip has a square cross sectional area of 50 × 50 μm.

### Tissue preparation

Mouse specimens, previously euthanized by CO_2_ gas, were collected on the day of testing from University College Dublin’s Biomedical Facility for indentation experiments. The specimens consisted of post-natal 56-day old mixed male and female mice. Mixed sex mice were used as it has been previously reported that gender has no effect on the dynamic compressive response of brain tissue^40^. In order to perform the indentation experiments, the brains were removed from the animals by making a midline incision through the skin across the top of the head to gain access to the skull. A second midline incision, moving anteriorly from the occipital condyle, was made through the skull using a scalpel. Two lateral incisions were then made at an anterior and posterior point of the midline incision so that the bone could be removed and allow access to the brain. The brain was then separated from the spinal cord and removed from the skull. Proceeding removal from the skull, the brains were kept hydrated with Phosphate Buffer Saline while the cortical surfaces of the cortex, cerebellum, and medulla were indented. The brains were not fixed to the platen as the weight of the brain was sufficient to prevent the organ from moving during application of the low-force indentations. Following indentations of these regions, the brains were sectioned along the sagittal sulci to gain access to the thalamus. This procedure was adopted to ensure the brain is kept as intact as possible for measuring the mechanical properties. The *in situ* protocol involved making a midline incision through the top of the skull preceding two lateral incisions across the skull, above the region of interest, allowing the bone to be removed and the brain to remain encased in the remaining skull. Each brain is continuously hydrated throughout the experiment with Phosphate Buffer Solution. All tests were completed within 6 hours post-mortem in order to reduce the amount of proteolysis and necrosis that has been previously shown to reduce the stiffness of the tissue^41^,^42^.

### Indentation protocol

Indentation tests were performed on *in vitro* (cortex, cerebellum, thalamus, and medulla, n = 120) and *in situ* (cortex and cerebellum, n = 60), mouse brains, up to 70 μm displacement, at velocities of 0.71, 2.14, and 4.28 mm/s, this encompasses a range of values which are both above and below the reported threshold for injury (10% strain, 10/s strain rate)^10^. To establish the contact point with the tissue, the indenter tip was brought into close proximity with the tissue. Once the indenter was sufficiently close to the tissue it was lowered in 2 μm increments until a force (<5 μN) was recorded from the indenter tip. If the force rose above 5 μN during this procedure, this region would not be measured and the indenter is moved to a new location for testing. No other preconditioning is performed on the sample as the sample is not preconditioned *in vivo*. The probe is then retracted 100 μm and the indentation is performed beyond 70 μm to ensure a constant velocity throughout indentation depth used for analysis. All tests were conducted at room temperature (~22 °C) and no tissue was reused after each indentation test due to the highly dissipative nature of brain tissue. The results were post-processed in MATLAB using a moving average filter over 10 data points. This procedure was performed to remove high frequency noise while keeping the indentation signal intact, this was ensured by monitoring the maximum indentation force at 70 μm before and after filtering so that the maximum force was not decreased.

The force-displacement curves were then imported into the inverse Finite Element model to determine the Neo-Hookean, Mooney-Rivlin, and Ogden material parameters. The Neo-Hookean model was chosen as it contains only one fitting parameter, *C*_*10*_, and many other hyperelastic models are extensions of the Neo-Hookean model, e.g. Mooney-Rivlin, and Yeoh. For the Neo-Hookean model the inverse Finite Element program is only required to fit one material parameter to the data allow the optimized value can be achieved with a greater confidence than those with more than one material parameter, giving an approximation for the value of the shear modulus. Once an approximation has been made for the shear modulus from the Neo-Hookean model, the force displacement data can then be applied to models containing more than one material parameter and their results be interpreted with greater confidence, as is in this case the Mooney-Rivlin, and Ogden hyperelastic material models. The Mooney-Rivlin model was chosen as it is an extension of the Neo-Hookean model to include the second strain invariant, *I*_2_, which describes the materials deviatoric deformation. Deviatoric deformation is required to fully describe a materials deformation. The Ogden model is used here due to its use in modelling the behavior of soft tissues by the biomechanics community^10^,^11^,^14^,^18^.

### Statistical analysis

ANOVA with a post hoc analysis was performed with each region *in vitro* and *in situ* to determine if a statistically significant difference exists between the brain regions based on the shear modulus values determined from the Neo-Hookean material model for n = 10 samples of each region at each velocity.

### Neo-Hookean hyperelastic model

The earliest hyperelastic material model based on the strain energy density function was put forward by Treloar in 1943 and is known as the Neo-Hookean material model^20^


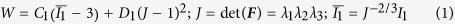


where *W* is the strain energy density, *C*_*1*_ = 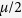, and *μ* is the shear modulus,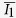 is the first strain invariant of the isochoric part of the Cauchy strain tensor, *I*_1_ is the first strain invariant of the Cauchy strain tensor, *λ*_*i*_ is the stretch ratio in the principal directions, and *D*_*1*_  = 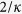, where *κ* is the bulk modulus of the material is assumed to be 10,000 × *μ*. This value for bulk modulus assumes slight compressibility of the material.

### Mooney-Rivlin hyperelastic model

The Mooney-Rivlin hyperelastic model was first proposed by Mooney (1940) as the most general form for a linear relationship between stress and strain in simple shear^43^. Rivlin used this model and the Neo-Hookean model in his earlier work on rubber elasticity. Suitable values of C_10_ and C_01_ can provide a better fit to experimental data than the Neo Hookean model


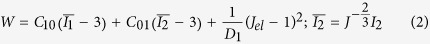


where *W* is the strain energy density, *μ* = 2(*C*_*10*_+*C*_*01*_) and *μ* is the shear modulus, 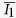 and 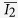 are the first and second strain invariants of the isochoric part of the Cauchy strain tensor, respectively *I*_2_ is the second strain invariant of the Cauchy strain tensor, *J*_*el*_ is the elastic volume ratio, and *D*_*1*_  = 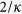, where *κ* is the bulk modulus of the material is assumed to be 10,000 × *μ*.

### Ogden hyperelastic model

Soft biological tissue under compression, tension, and shear is often modelled well by the Ogden model^11^,^14^. In this study the Ogden model is extended to indentation using the inverse Finite Element method. The Ogden strain energy potential is expressed in terms of the principal stretches. The Abaqus formulation is of the form:


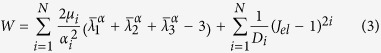


where *W* is the strain energy density, *μ* is the shear modulus, 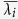 are the principal stretches of the strain invariants of the isochoric part of the Cauchy strain tensor, *λ*_*i*_ are the stretch ratio in the principal directions, *D*_*1*_ = 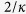, where *κ* is the bulk modulus of the material is assumed to be 10,000 × *μ*, and *α* is a material constant that is a real number, positive or negative^44^.

### Inverse Finite Element analysis

Initial estimates for the material parameters are input to the inverse Finite Element analysis program (ABAQUS, Dassault Systemes, MATLAB, Mathworks) to determine the actual material parameters for the Neo-Hookean, Mooney-Rivlin, and Ogden hyperelastic models. Taking advantage of the model’s symmetry a one-quarter model was used. The model consisted of a rigid indenter tip geometry, modeled at one-quarter of the indenter geometry in Fig. 1b, consisting of 1006 linear quadrilateral R3D4 elements. The rigid indenter tip was initially in contact with a deformable cylinder, with a radius of 1000 μm and height of 1500 μm, consisting of 8160 linear hexahedral C3D8RH reduced integration, hybrid elements, which represents a sample of brain tissue. A fixed boundary condition was applied to the bottom face of the cylinder, and a displacement boundary condition of 70 μm was applied to the indenter tip. Once the initial estimate for material parameters has been input to the analysis, an ABAQUS finite element simulation of the experiment is performed. The force-displacement data from the indenter tip is extracted from the simulation result file and compared with the force-displacement curves from experiment. Once the data with the best fit has been determined, using the sum of absolute differences method, the analysis terminates and the material parameters providing the best fit between the experiment and simulation is found. This process is repeated for each region and velocity.

## Additional Information

**How to cite this article**: MacManus, D. B. *et al.* Mechanical characterization of the P56 mouse brain under large-deformation dynamic indentation. *Sci. Rep.* **6**, 21569; doi: 10.1038/srep21569 (2016).

## Figures and Tables

**Figure 3:**
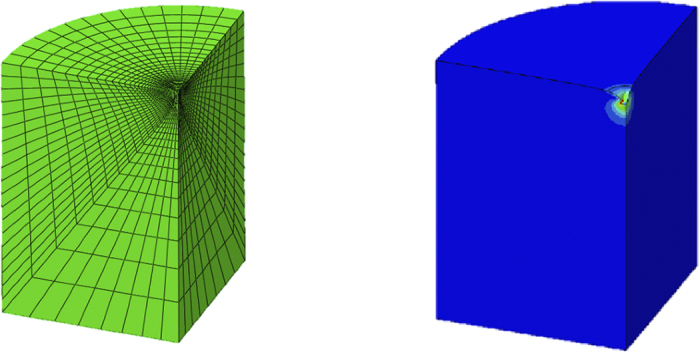
One-quarter Finite Element Model of Indentation Experiment on Mouse brain tissue in the reference (green) and deformed configurations which was used in the inverse Finite Element method to characterize the dynamic mechanical properties of mouse brain tissue.

**Table 1:** Presents regional brain tissue material parameters for the Neo-Hookean, Mooney-Rivlin and Ogden models, used to model brain tissue under mechanical loads, where C_10_, C_01_ are related to the shear modulus, μ is the shear modulus, and α is a nonlinear parameter.

Velocity (mm/s)	Neo-Hookean	Mooney-Rivlin		Ogden	
Cerebral Cortex	C_10_	C_10_	C_01_	μ_0_	α
0.71	1.11 ± 0.06	0.90 ± 0.26	0.22 ± 0.12	2.09 ± 0.16	0.1 ± 0.83
R^2^	0.999	0.999		0.999	
2.14	2.63 ± 0.16	2.19 ± 0.12	0.34 ± 0.05	5.11 ± 0.31	1.23 ± 0.49
R^2^	0.999	0.998		0.999	
4.28	3.39 ± 0.64	1.62 ± 0.27	1.46 ± 0.41	6.44 ± 1.16	0.38 ± 0.63
R^2^	0.998	0.976		0.999	
Cerebellum	**C**_**10**_	**C**_**10**_	**C**_**01**_	**μ**_**0**_	**α**
0.71	0.67 ± 0.11	0.90 ± 0.26	0.22 ± 0.12	1.27 ± 0.20	0.26 ± 0.49
R^2^	0.999	0.999		0.999	
2.14	1.18 ± 0.36	2.19 ± 0.12	0.34 ± 0.05	2.33 ± 0.70	1.54 ± 0.69
R^2^	0.999	0.998		0.999	
4.28	1.19 ± 0.17	1.62 ± 0.27	1.46 ± 0.41	2.25 ± 0.32	0.10 ± 0.02
R^2^	0.999	0.999		0.999	
Thalamus	**C**_**10**_	**C**_**10**_	**C**_**01**_	**μ**_**0**_	**α**
0.71	0.72 ± 0.06	0.52 ± 0.06	0.16 ± 0.01	1.40 ± 0.11	1.19 ± 0.68
R^2^	0.998	0.997		0.998	
2.14	0.74 ± 0.1	2.56 ± 0.11	0.15 ± 0.02	1.45 ± 0.17	0.89 ± 0.16
R^2^	0.998	0.998		0.999	
4.28	0.81 ± 0.15	0.50 ± 0.12	0.20 ± 0.03	1.51 ± 0.27	0.13 ± 0.01
R^2^	0.999	0.998		0.999	
Medulla Oblongata	**C**_**10**_	**C**_**10**_	**C**_**01**_	**μ**_**0**_	**α**
0.71	0.33 ± 0.08	0.15 ± 0.04	0.16 ± 0.06	0.63 ± 0.01	0.29 ± 0.28
R^2^	0.997	0.999		0.998	
2.14	0.51 ± 0.20	0.30 ± 0.09	0.13 ± 0.02	0.74 ± 0.18	−2.15 ± 0.08
R^2^	0.986	0.996		0.999	
4.28	0.66 ± 0.23	0.53 ± 0.23	0.11 ± 0.004	1.17 ± 0.39	−1.07 ± 0.03
R^2^	0.989	0.991		0.994	
Cerebral Cortex (*In Situ*)	**C**_**10**_	**C**_**10**_	**C**_**01**_	**μ**_**0**_	**α**
0.71	2.29 ± 0.14	1.66 ± 0.13	0.52 ± 0.01	4.53 ± 0.26	1.73 ± 0.14
R^2^	0.999	0.998		0.999	
2.14	3.30 ± 0.85	1.88 ± 0.81	0.97 ± 0.29	6.32 ± 1.55	1.11 ± 1.57
R^2^	0.995	0.996		0.997	
4.28	3.76 ± 0.81	2.42 ± 0.82	1.10 ± 0.03	7.21 ± 1.48	0.59 ± 0.80
R^2^	0.999	0.999		0.999	
Cerebellum (*In Situ*)	**C**_**10**_	**C**_**10**_	**C**_**01**_	**μ**_**0**_	**α**
0.71	0.95 ± 0.29	0.64 ± 0.33	0.25 ± 0.09	1.74 ± 0.56	0.47 ± 2.14
R^2^	0.998	0.997		0.997	
2.14	1.08 ± 0.37	0.94 ± 0.37	0.11 ± 0.04	2.09 ± 0.69	0.93 ± 0.37
R^2^	0.997	0.998		0.998	
4.28	1.13 ± 0.95	0.99 ± 0.94	0.11 ± 0.01	2.02 ± 1.62	−0.29 ± 1.21
R^2^	0.990	0.991		0.993	

Hyperelastic Material Parameters of P56 Murine Brain Regions.
